# Use of concept mapping to inform a participatory engagement approach for implementation of evidence-based HPV vaccination strategies in safety-net clinics

**DOI:** 10.1186/s43058-024-00607-7

**Published:** 2024-06-26

**Authors:** Jennifer Tsui, Michelle Shin, Kylie Sloan, Thomas I. Mackie, Samantha Garcia, Anne E. Fehrenbacher, Benjamin F. Crabtree, Lawrence A. Palinkas

**Affiliations:** 1 Department of Population and Public Health Sciences, Keck School of Medicine, University of Southern California, 1441 Eastlake Ave, Los Angeles, CA 90033 USA; 2 Norris Comprehensive Cancer Center, University of Southern California, Los Angeles, CA USA; 3 Department of Child, Family, and Population Health Nursing, School of Nursing, University of Washington, Seattle, WA USA; 4 Department of Social and Behavioral Sciences, School of Nursing, University of California, San Francisco, USA; 5 School of Public Health, SUNY Downstate Health Sciences University, Brooklyn, NY USA; 6 Department of Family Medicine and Community Health, Rutgers Robert Wood Johnson Medical School, Rutgers the State University of New Jersey, New Brunswick, NJ USA; 7 Rutgers Cancer Institute of New Jersey, Rutgers the State University of New Jersey, New Brunswick, NJ USA; 8 Herbert Wertheim School of Public Health and Longevity Science, University of California San Diego, La Jolla, CA USA

**Keywords:** HPV vaccination, Concept mapping, Evidence-based strategies, Implementation context, Health equity, Safety-net settings

## Abstract

**Background:**

Multiple evidence-based strategies (EBS) for promoting HPV vaccination exist. However, adolescent HPV vaccination rates remain below target levels in communities at high risk for HPV-associated cancers and served by safety-net clinics. Participatory engaged approaches are needed to leverage the expertise of community and clinical partners in selecting EBS relevant to their local context. We engaged concept mapping as a method to inform the adoption and adaptation of EBS that seeks to empower implementation partners to prioritize, select, and ultimately implement context-relevant EBS for HPV vaccination.

**Methods:**

Using 38 EBS statements generated from qualitative interviews and national HPV vaccine advocacy sources, we conducted a modified concept mapping activity with partners internal to safety-net clinics and external community members in two study sites of a larger implementation study (Greater Los Angeles and New Jersey), to sort EBS into clusters and rate each EBS by importance and feasibility for increasing HPV vaccination within safety-net clinics. Concept mapping findings (EBS statement ratings, ladder graphs and go-zones) were shared with leaders from a large federally qualified health center (FQHC) system (focusing on three clinic sites), to select and implement EBS over 12 months.

**Results:**

Concept mapping participants (n=23) sorted and rated statements, resulting in an eight-cluster solution: 1) Community education and outreach; 2) Advocacy and policy; 3) Data access/quality improvement monitoring; 4) Provider tracking/audit and feedback; 5) Provider recommendation/communication; 6) Expanding vaccine access; 7) Reducing missed opportunities; and 8) Nurse/staff workflow and training. The FQHC partner then selected to intervene on eight of 17 EBS statements in the “go-zone” for action, with three from “reducing missed opportunities,” two from “nurse/staff workflow and training,” and one each from “provider tracking/audit and feedback,” “provider recommendation/communication,” and “expanding vaccine access,” which the research team addressed through the implementation of three multi-level intervention strategies (e.g., physician communication training, staff training and workflow assessment, audit and feedback of clinic processes).

**Conclusions:**

Concept mapping provided a powerful participatory approach to identify multilevel EBS for HPV vaccination relevant to the local safety-net clinic context, particularly when several strategies exist, and prioritization is necessary. This study demonstrates how a clinic system benefited directly from the ratings and prioritization of EBS by multilevel clinic and community partners within the broader safety-net clinic context to identify and adapt prioritized solutions needed to advance HPV vaccine equity.

Contributions to the literature

- Our study demonstrates how concept mapping can respond to the complexities of internal and external contexts for implementation by assisting in the prioritization and selection of evidence-based strategies (EBS) by key community partners.
- Clinic and community members were consistent in their high prioritization of provider- and clinic-team focused EBS and increasing access to vaccines, over community outreach and data accessibility.
- Connecting safety-net system providers to concept mapping ratings of EBS strategies can be a powerful participatory engagement approach to inform adoption and implementation of EBS within local clinical contexts.

## Background

Human papillomavirus (HPV) vaccines were introduced nearly two decades ago, but uptake among adolescents remains suboptimal [1, 2]. Despite its effectiveness [3–6] and widespread availability, only 63% of adolescents ages 13-17 years were up to date with HPV vaccination in the United States (US) in 2022 [7], below the 80% HPV vaccination goal outlined in Healthy People 2030 [8]. Importantly, for the first time in several years, HPV vaccine initiation rates among Medicaid beneficiaries declined from previous years [7]. Failure to meet target HPV vaccination rates among adolescents in communities served by safety-net clinics is of particular concern, as these are the same communities that experience a disproportionate burden of cervical and other HPV-associated cancers. The implementation of evidence-based strategies (EBS) to improve HPV vaccination within safety-net clinics is critical to addressing long-term cancer inequities [9–12].

Multiple EBS to support implementation of HPV vaccination emerged in recent years, which include targeting internal (e.g., provider, clinic team, health system-level) and external (e.g., community, policy) contexts relevant to safety-net clinics, and currently endorsed by the National HPV Vaccine Roundtable and other advocacy and professional organizations [13, 14]. However, the prioritization and feasibility of the implementation of EBS within safety-net settings in alignment with context and fit remains understudied [15]. Strategies are needed to assist clinic and community partners who serve adolescents in safety-net settings to prioritize and select EBS for HPV vaccination, through a user-centered design approach [16], to accommodate the primarily low-resource settings that serve communities with disproportionately greater social needs [17]. This is even more important following missed doses in adolescent HPV vaccination due to COVID-19 pandemic disruptions [7, 18] and given the potential to leverage new local policy-level immunization strategies as a result of COVID-19 vaccination efforts [19].

Recent advances in the development [20], selection [21], implementation [22], and reporting [23] of implementation strategies offers promise in expediting the translation of evidence into practice, including for HPV vaccination. Implementation strategies, defined as “methods or techniques used to enhance the adoption, implementation, and sustainability of a clinical program or practice,” are advanced largely to bridge the quality chasm in healthcare [24]. However, the breadth of available implementation strategies continues to challenge the field of implementation science requiring investment in methods that can elicit multilevel and EBS that align with a specific delivery system setting [25, 26]. Furthermore, equitable implementation requires explicit attention to the culture, history, values, and needs of the community and integration of these factors to optimize fit, inform adaptation, and ensure sustainability [27]. For example, our prior work with American Indian/Alaska Native, Hispanic/Latino and Chinese parents of unvaccinated children identified relationships with adolescent children, exposure to misinformation through community channels, and historical community experiences that contribute to medical mistrust vary across communities and impact how communities engage with primary care providers and prefer to receive vaccine information recommendation, and decision-making [28, 29]. These community and setting specific factors can influence the prioritization of specific EBS over others.

Concept mapping offers an approach to engage local communities in the task of eliciting and identifying selection of implementation strategies for HPV vaccination that can increase likelihood of successful outcomes and advance health equity [30, 31]. Concept mapping is a mixed methods approach in participatory research, which involves direct engagement and collaboration with those affected by a specific topic for the purpose of action or change [32, 33]. Traditionally, concept mapping has been used to conceptualize and prioritize local knowledge, needs and values from community members who are directly impacted by the particular topic to form the basis for research and planning [32–35]. Prior concept mapping studies relevant to HPV vaccination have mainly focused on the perceptions and priorities of clinic and community members on adolescent immunization, HPV vaccination efforts, and facilitators and barriers to implementation of EBS for vaccination [36–38]. The potential for concept mapping to inform the selection and tailoring of strategies [21, 39] to fit local contexts and settings [40, 41] have been demonstrated for other disease topics, but not yet used for HPV vaccination. Thus, concept mapping techniques may be optimal to select and tailor evidence-based strategies for HPV vaccination, where multiple evidence-based strategies (EBS) for promoting HPV vaccination exist.

In this study, we aim to describe how concept mapping was applied as a tool to rate existing HPV EBS for both the internal and external factors in local safety-net clinic contexts, and ultimately to support a participatory engaged process for selection and equitable implementation of HPV vaccination EBS within a large FQHC setting [42]. Many implementation frameworks highlight that both internal and external contextual factors influence implementation and sustainment [43, 44]. While prior studies demonstrate that internal factors, those characteristics within clinics serving adolescents (e.g., provider characteristics, organizational leadership), and external factors, those characteristics outside of the organization (e.g., state-level policies), contribute to complex and often competing influences in HPV vaccination strategies [15, 19, 45], few studies focus on participatory approaches and effective tools that can be utilized to facilitate the selection of strategies that address implementation barriers. In this concept mapping study, we examine how community members both internal and external to safety-net clinics view the importance and feasibility of strategies to inform prioritization, selection, and fit for HPV vaccine improvement within the local context. By providing strategies across internal and external contexts, opportunities exist to advance multilevel strategies posited widely in implementation science frameworks as critical to supporting the implementation and sustainment of evidence-based practices [44, 46].

## Methods

### Study overview

We conducted modified concept mapping in Greater Los Angeles (LA), California (CA) and New Jersey (NJ) with purposively sampled participants, who were internal (e.g., clinic leaders/administrators, providers/staff [MD, MA]) and external (e.g., advocates, policy representatives) to safety-net clinics, who previously participated in qualitative interviews about their experiences with EBS in safety-net settings as part of a larger multi-year study to identify factors associated with implementation of EBS for HPV vaccination [15]. Concept mapping was subsequent step to the qualitative interviews conducted in the larger study. Primary findings from these qualitative interviews on clinic and community member perspectives of EBS for HPV vaccination [15] as well as perspectives on payer and policy influences [19], the impact of the COVID-19 pandemic on EBS [47], and school-based opportunities for EBS implementation [17] have been previously described. In brief, we used the Practice Change Model (PCM), a conceptual model used to understand the critical elements of practice change and implementation of evidence-based practices, to guide interviews to examine internal and external practice-based factors that impact adoption of EBS for HPV vaccination and the interrelationships among these factors [48]. As described in our previous work [15], PCM domains included: (1) Motivation to implement EBS for HPV vaccination in clinic settings, (2) Existing resources to promote EBS for HPV vaccination in clinic settings, (3) Clinics’ motivators for implementing EBS or how participants serve as outside motivators in clinic settings, (4) Noted opportunities for change within clinics to implement or promote EBS, and interrelationships across domains 1 through 4. Thus, strategies identified from qualitative interviews and described below for concept mapping are relevant to internal resources and external motivators for HPV vaccination in primary care safety-net settings.

**Fig. 1:**
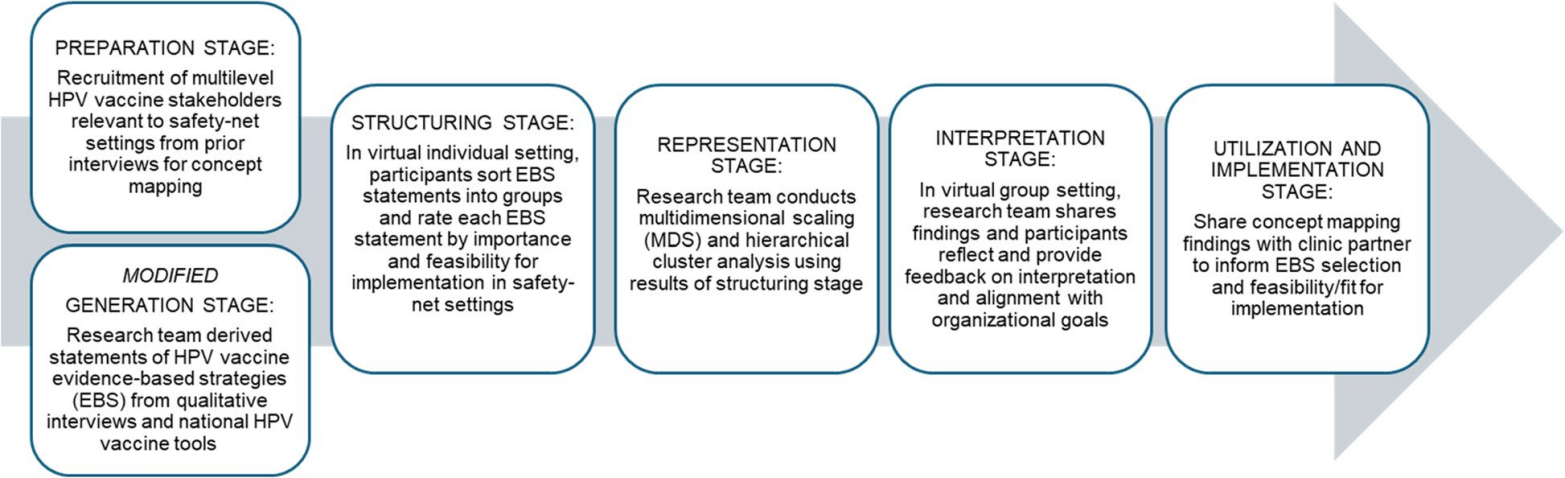
Concept mapping process for prioritization and implementation of evidence-based HPV vaccination strategies

We followed Green and colleagues [31] six main phases for concept mapping: (1) preparation, (2) generation, (3) structuring, (4) representation, (5) interpretation, and (6) utilization. While results of concept maps are traditionally used to guide measurement and constructs development among a group of partners and members [49], we used concept mapping to facilitate community and clinical partners in prioritizing and selecting EBS for HPV vaccination relevant to their local context (Fig. 1).

### Concept mapping procedures

#### Preparation stage

Recruitment for the concept mapping phase began in April 2022, 3-15 months following individual qualitative interviews. Participants, providers, clinic staff, clinic and system leaders, policy and payer representatives, and local and state HPV vaccine advocates, who agreed to be re-contacted for following their qualitative interviews the concept mapping phase of the study were contacted by email. Of the 58 participants from qualitative interviews, ten were no longer at their prior organizations. In June and July 2022, ten additional participants were recruited during state and regional HPV vaccine coalition meetings based on similar experiences of being HPV vaccine champions. Upon recruitment, all invited participants (n=58) received details on the purpose of concept mapping activity, how to access the Groupwisdom^TM^ web-based platform [35], and instructions for sorting and rating EBS statements. Participants were sent up to three email reminders to complete concept mapping. This study was approved by the University of Southern California Institutional Review Board.

#### Modified generation stage

Concept mapping typically involves brainstorming session in which different participants meet to identify key topics for discussion and formulate statements summarizing these concepts [50]. In this study, prior to contacting participants for concept mapping, EBS for HPV vaccination were derived from qualitative data and advocacy sources and guidelines. We initially identified 64 EBS relevant to HPV vaccination from qualitative interviews and 20 additional EBS for HPV vaccination from existing national sources and guidelines, including the *Clinician and Health System Action Guides* from the National HPV Vaccination Round Table and *Evidence-Based Cancer Control Programs (EBCCP)* from the National Cancer Institute [13, 51]. After eliminating duplicate strategies or similar strategies across target levels (i.e., use of EHR for physician reminders indicated at the health system and provider target levels), 40-50 EBS statements were sent to HPV vaccine partners and advocates in both states for member-checking and further distillation. HPV vaccine partners and advocates who reviewed and further distilled statements included 4 local/state representatives American Cancer Society, HPV Vaccine Roundtable, community-based organization and research consultants, who did not participate in later steps of the concept mapping activity. Member checking and group consensus with the research team resulted in a final list of 38 EBS for HPV vaccination inputted as statements for concept mapping into the Groupwisdom^TM^ web-based platform.

This brainstorming approach deviated from other concept mapping studies (e.g., simultaneous brainstorming with sorting and rating across participants) to maximize the use of expertise among the purposive sample of HPV vaccine champions, who provided real-time field observation of HPV vaccination within safety-net settings during the pandemic. Ultimately, our sampling approach sought to elicit a comprehensive list of EBS for sorting and rating, including those that may have otherwise been unknown within a specific healthcare system or community setting.

#### Structuring Stage

Upon email recruitment between April-July 2022, participants were asked to log into the self-administered Groupwisdom^TM^ platform and complete two phases of concept mapping: (1) sorting and rating and (2) interpretation. Participants first received the following focus prompt: *“To increase HPV vaccination among adolescents (ages 9-13 years) in healthcare settings serving medically underserved communities, we can do the following….”* to review the 38 EBS statements identified in the prior phase. Each EBS statement appeared as a “card” on the screen. Participants were asked to sort the 38 statements by dragging and dropping each card onto the right side of the screen to create “piles.” Participants were given the following instructions for sorting: “In this activity, you will categorize statements into piles according to your view of their meaning. To do this, you will sort each statement into piles in a way that makes sense to you. Group the statements on how similar in meaning they are to one another.” Once the participants moved all the statements into a pile, they were instructed to name each pile to describe its contents.

Following the sorting activity, participants were asked to rate each statement by importance and feasibility for increasing HPV vaccination in their organization or region on a 4-point scale. The 4-point importance scale ranged from “not important at all [1]” to “extremely important [4].” The 4-point feasibility scale ranged from “not doable at all right now [1]” to “extremely doable right now [4].” The self-administered sorting and rating task was intended to take participants approximately 20-30 minutes total to complete. We piloted the Groupwisdom^TM^ platform with study team members and advocacy partners to ensure clarity of instructions and statements. All concept mapping participants received a $50 gift card upon completion of both sorting and rating phases.

#### Representation stage

Using the Groupwisdom^TM^ concept mapping platform, we conducted multidimensional scaling (MDS) and hierarchical cluster analysis to characterize how the named piles were clustered by participants [31, 49]. A point map was generated to position each EBS for HPV vaccination on a two-dimensional map with four poles where strategies located close to each other carried a similar meaning and elements further apart were less related. The coordinates of the point map were then used to conduct hierarchical cluster analysis. A similarity matrix was created to examine overall prioritization of EBS as well as configurations for specific participant groups.

The analysis produced cluster maps (weighted and unweighted), ladder graphs, and go-zone maps (e.g., most important and most feasible strategies). The research team compared maps of seven, eight, and nine clusters before making a group decision based on interpretability, operationally defined as reaching consensus that creating an additional cluster would not improve the meaningfulness of the data, to finalize using the map of eight clusters. The 7-cluster map combined “provider tracking/audit and feedback” statements with the “Data/QI Monitoring” cluster which was a distinct cluster in the final 8 cluster map. The 9-cluster map had a separate cluster of “community/cultural engagement” statements that were combined with the “community education and outreach” cluster in the final 8 cluster map. Data from participants’ naming of statement piles and an examination of the individual statements within each cluster were used to inform the research team’s final naming of the eight clusters. For sensitivity analyses, we compared common and divergent ratings across internal (e.g., providers) and external (e.g., advocates) participant groups and across the two regions (LA and NJ) by creating and comparing cluster maps and ladder graphs for each of the four subgroups.

We generated a go-zone map for all participants combined. The go-zone map displayed the 38 statements as a function of their importance and feasibility. The top left quadrant of the go-zone map (Fig. 4) is the “go zone” and where the statements most highly rated as “important” and “feasible” are located.

#### Interpretation stage

All participants who completed the sorting and rating phases of concept mapping were contacted by email in September 2022 to participate in a one-hour virtual group interpretation meeting via Zoom. Upon recruitment for this phase, participants received a handout with preliminary findings via email, which included the eight-cluster map, ladder graphs, and go-zone map comprised of all responses in aggregate. At the interpretation meeting, participants were asked to reflect and share their feedback on the concept mapping activity results (e.g., explanation of the cluster map, ladder graphs, and go-zone map). A discussion guide, constructed by the research team, was used for the meeting to focus on: 1) participants’ overall thoughts about the final eight clusters that resulted from the sorting activity, 2) reactions to the relative ratings for importance and feasibility of strategy clusters, and 3) thoughts on how the go-zone map aligned with their organizations’ current approaches for HPV vaccination. The 60-minute session was recorded and then transcribed by a third-party transcription service. Two research team members then read through the transcript for a content analysis of overall themes, structured around the Practice Change Model which guided the larger overall study, and key areas of divergence among participants, if any. All interpretative session participants were provided a $50 gift card upon completion.

#### Utilization and implementation stage

We shared the concept mapping results with system leaders from a large multi-site FQHC system in LA and physician and clinic champions from three clinic sites within the FQHC. We conducted an initial meeting in August 2022 with FQHC system leaders (EHR lead analyst, health equity research partnership lead, chief of pediatrics) and clinic leaders (e.g., physician champions, clinic directors, non-clinician staff members) from clinic sites. Research team members shared the eight-cluster map, ladder graphs, and go-zone map to inform selection of HPV vaccination EBS for implementation. Clinic leaders and champions and research team members discussed strategies prioritized from concept mapping results as well as the current clinical context and strategies used within the FQHC. Then together, the group (researchers, system leaders and champions) selected strategies during the presentation, which were finalized with physician champions at each of the three clinics in October 2022 and then implemented at each of the three clinics between December 2022 and August 2023. We use the Standards for Reporting Implementation Studies (StaRI) checklist to describe the implementation strategy (i.e., stakeholder engagement, facilitation of prioritizing HPV vaccination practices) the intervention (i.e., concept mapping) that is being implemented and described in this manuscript.

## Results

### Sample characteristics

A total of 23 participants, among 58 total invited to participate, completed the concept mapping sorting and rating activity (Table 1). Among these, 10 (43%) participants were internal clinic members (clinic leaders *n*=4; providers *n*=4; clinic staff *n*=2) and 13 (57%) participants were external community members (policy representatives (*n*=3); advocates (i.e., *n*=10)). Advocates included representatives from state HPV Vaccine Roundtables, local and county HPV vaccine coalitions, the American Cancer Society, and community-based organizations and partnerships focused on vaccine promotion. Participants were from two regions (LA=13; NJ=10).

**Table 1:** Concept Mapping Participant Characteristics from Sorting and Rating Phase (*n*=23)

	n	%
**Participant’s Primary Role**		
Provider with direct patient contact (MD, NP, etc.)	4	17.4
Clinic staff (MA, RN, etc.)	2	8.7
Clinic leader/administrator	4	17.4
Policy	3	13.0
Advocacy	10	43.5
**Region**		
Los Angeles	13	43.5
New Jersey	10	56.5
**Race/ethnicity**^**a**^		
Hispanic/Latino/a	7	28
Black or African American	5	20
Asian or Asian American	3	12
White	10	40
**Gender identity**		
Female	18	78.3
Male	5	21.7
**Age**		
20-29	3	13.0
30-39	6	26.1
40-49	6	26.1
50-59	5	21.7
60-69	3	13.0

^a^Participants were able to select multiple race groups

### Cluster descriptions

**Fig. 2:**
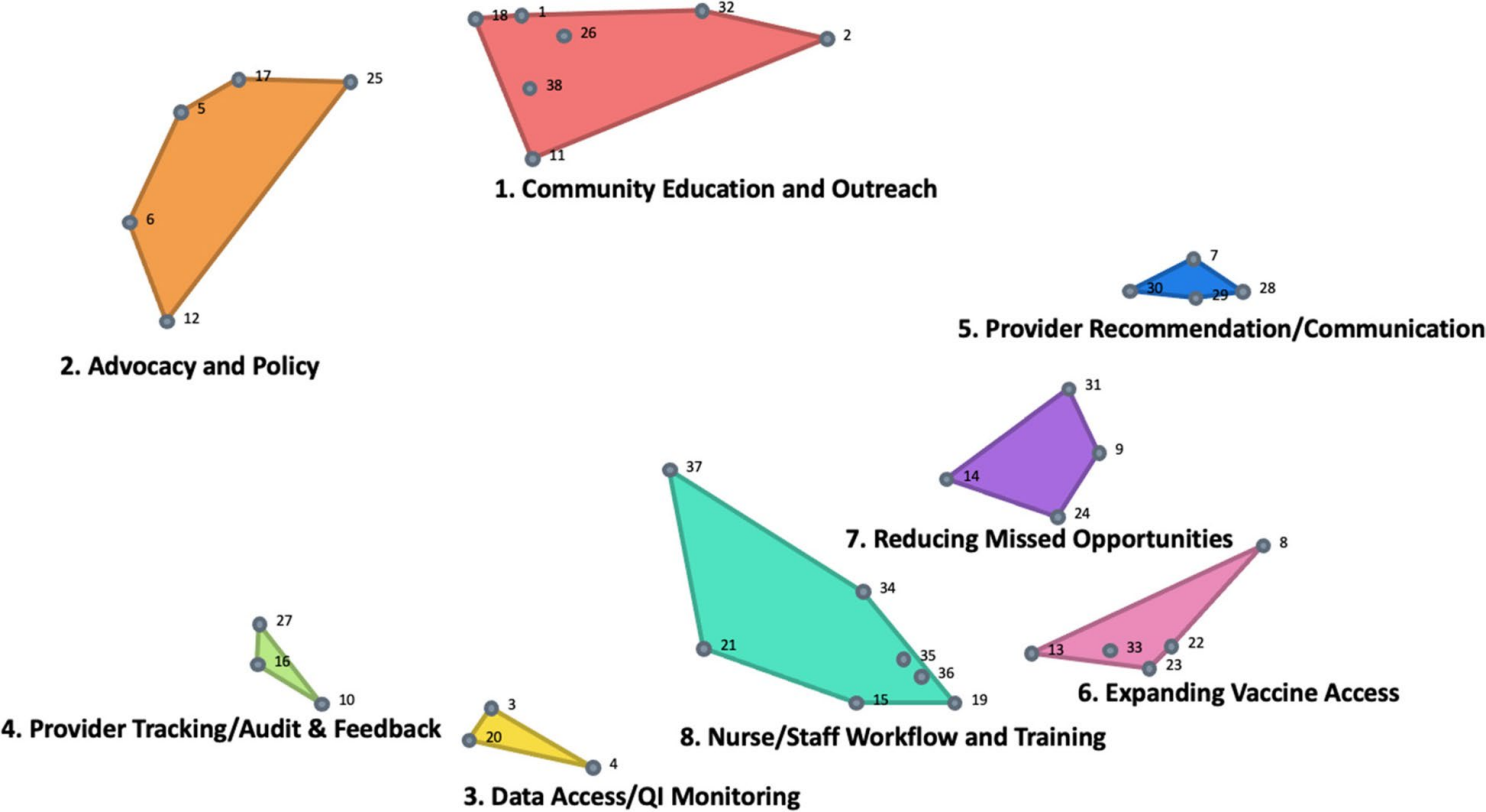
Cluster map of evidence-based strategies (EBS) for implementation in safety-net settings to increase HPV vaccination

Eight clusters of EBS for HPV vaccination emerged from the sorting phase: 1) Community education and outreach; 2) Advocacy and policy; 3) Data access/quality improvement (QI) monitoring; 4) Provider tracking/audit and feedback; 5) Provider recommendation/communication; 6) Expanding vaccine access; 7) Reducing missed opportunities; and 8) Nurse/staff workflow and training (Fig. 2). To ensure comparability, we used the overall cluster solution and examined differences in importance and feasibility ratings overall and then for each subgroup (e.g., internal clinic members, external community members, LA, NJ). See Table 2 for the full list of strategies within each of the eight clusters.

**Table 2:** Clusters and Individual EBS with Means for Importance and Feasibility Ratings within Safety-Net Settings

Statement #	Statement	Importance (Mean: Scale 1-4)	Feasibility (Mean: Scale 1-4)
Cluster 1: Community Education and Outreach		3.19	2.54
1	Have community liaisons/promotores go door-to-door to raise awareness about HPV vaccines	2.83	2.13
2	Provide in-language education via cultural liaisons to improve trust in HPV vaccine & safety	3.65	2.61
11	Provide resources for school-based clinics/school districts to educate adolescents about HPV vaccine	3.39	3.13
18	Engage hyper-local/ethnic media to raise awareness in immigrant communities	3.39	2.61
26	Engage adolescent peer leaders to use social platforms to promote HPV vaccines	3.26	2.73
32	Print and distribute HPV awareness posters through local channels (FQHCs, school nurses) in multiple languages	3.26	2.91
38	Have community liaisons/promotores go door-to-door to administer HPV vaccines	2.57	1.65
Cluster 2: Advocacy and Policy		3.03	2.7
5	Encourage intersectoral collaboration within local coalitions to share best practices	3.13	2.65
6	Engage advocacy groups to conduct quality improvement projects within clinics	3.09	2.61
12	Invite vaccine experts/pharmaceutical companies to educate providers about communication strategies	2.78	2.87
17	Establish a local community advisory committee with multiple stakeholders to get buy-in on clinic projects to improve HPV vaccine rates	3.26	2.65
25	Host a community townhall/webinar series with experts to raise HPV vaccine awareness	2.87	2.7
Cluster 3: Data Access/QI Monitoring		3.42	3.1
3	Ensure bi-directionality between state immunization registry & clinic record system	3.48	3.0
4	Have clinics routinely access state immunization registry for up-to-date HPV vaccine records	3.3	3.13
20	Monitor/report/provide to clinic teams on their site-specific HPV vaccine rates	3.48	3.17
Cluster 4: Provider Tracking/Audit & Feedback		3.3	3.32
10	Track HPV vaccine-specific rates separately from other adolescent vaccines (e.g. Tdap)	3.17	3.3
16	Monitor/report/provide feedback to individual providers on their HPV vaccine rates	3.48	3.26
27	Routinely compare HPV vaccine-specific rates to other adolescent vaccine rates	3.26	3.39
Cluster 5: Provider Recommendation/Communication		3.68	3.78
7	Provide parents/adolescents with HPV vaccine educational materials during clinic visit	3.35	3.74
28	Provide parents/adolescents an opportunity to ask about HPV vaccine during visits	3.83	3.96
29	Start the conversation about HPV vaccine at age 9 with parents	3.73	3.57
30	Focus on HPV vaccines as a cancer prevention vaccine and not on sexual transmission	3.83	3.87
Cluster 6: Expanding Vaccine Access		3.57	3.22
8	Promote co-administration of HPV vaccination at flu/COVID-19 vaccine events on weekends/after-hours	3.52	2.87
13	Schedule appointments for follow-up doses before the adolescent leaves the clinic	3.74	3.57
22	Provide opportunities for vaccination-only/nurse-visit for HPV vaccination	3.61	3.3
23	Offer weekend or evening clinic hours for HPV vaccination	3.35	2.91
33	Provide opportunities for HPV vaccinations during all visits including sick/urgent care visits	3.65	3.45
Cluster 7: Reducing Missed Opportunities		3.47	3.59
9	Normalize HPV vaccination as "opt-out" event versus "optional vaccine" during clinical encounter	3.65	3.57
14	Encourage providers/staff to recommend HPV vaccine by bundling with other vaccines	3.65	3.74
24	Encourage providers/staff to recommend HPV vaccines assuming the parent/adolescents will receive it (announcement approach)	3.61	3.65
31	Encourage providers/staff give personal examples of choosing HPV vaccine for family/friends	2.96	3.39
Cluster 8: Nurse/Staff Workflow and Training		3.65	3.32
15	Set up EHR alerts for providers/staff to vaccinate adolescents due/overdue for the HPV vaccine	3.78	3.48
19	Remind parents/adolescents of due/overdue HPV vaccine doses via patient recall system	3.65	3.39
21	Appoint clinic champion/workgroup to implement HPV vaccine improvement strategies	3.52	2.87
34	Utilize pre-clinic meetings/huddles to ensure all team members know their roles in the vaccine workflow	3.43	3.35
35	Clinic staff can pre-chart to see if the scheduled adolescent is due/overdue for HPV vaccine before each visit	3.78	3.3
36	Start/continue using HPV vaccine standing orders so trained staff can order/administer	3.74	3.39
37	Provide training for all clinic staff on HPV vaccine recommendation strategies to ensure consistent messaging	3.61	3.43

### Cluster ratings

#### Combined

**Fig. 3:**
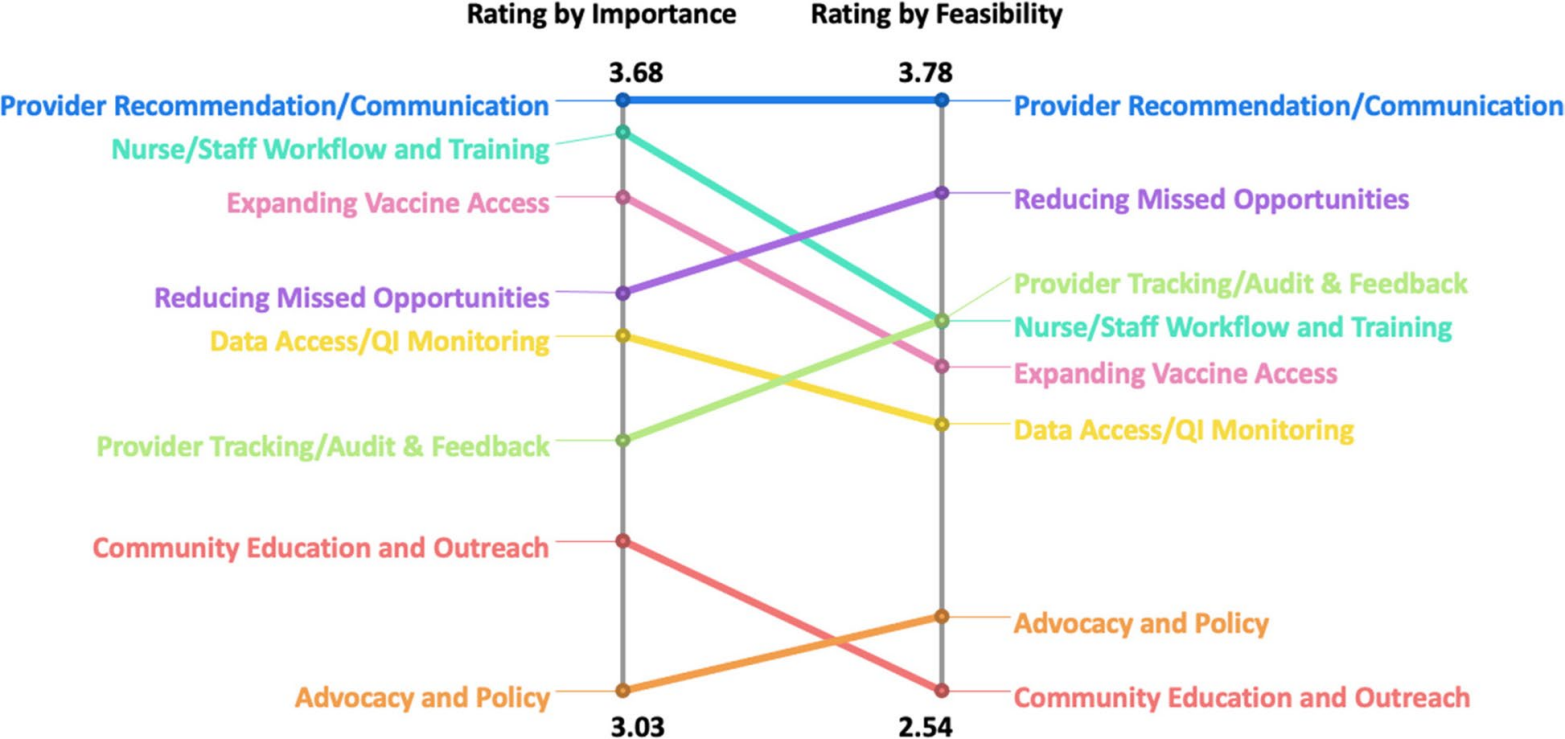
Ladder graph of comparison of importance and feasibility ratings for each cluster

Clusters and mean importance and feasibility ratings for individual EBS (e.g., statements) are listed in Table 2. “Provider recommendation/communication” (mean=3.68), “nurse/staff workflow and training” (mean=3.65), and “expanding vaccine access” (mean=3.57) were rated as the most important clusters of strategies. While “provider recommendation/communication” was also rated as most feasible (mean=3.78), the next three most feasible strategies were “reducing missed opportunities” (mean=3.59), “provider tracking/audit and feedback” (mean=3.32), and “nurse/staff workflow and training” (mean=3.32) (Fig. 3).

**Fig. 4:**
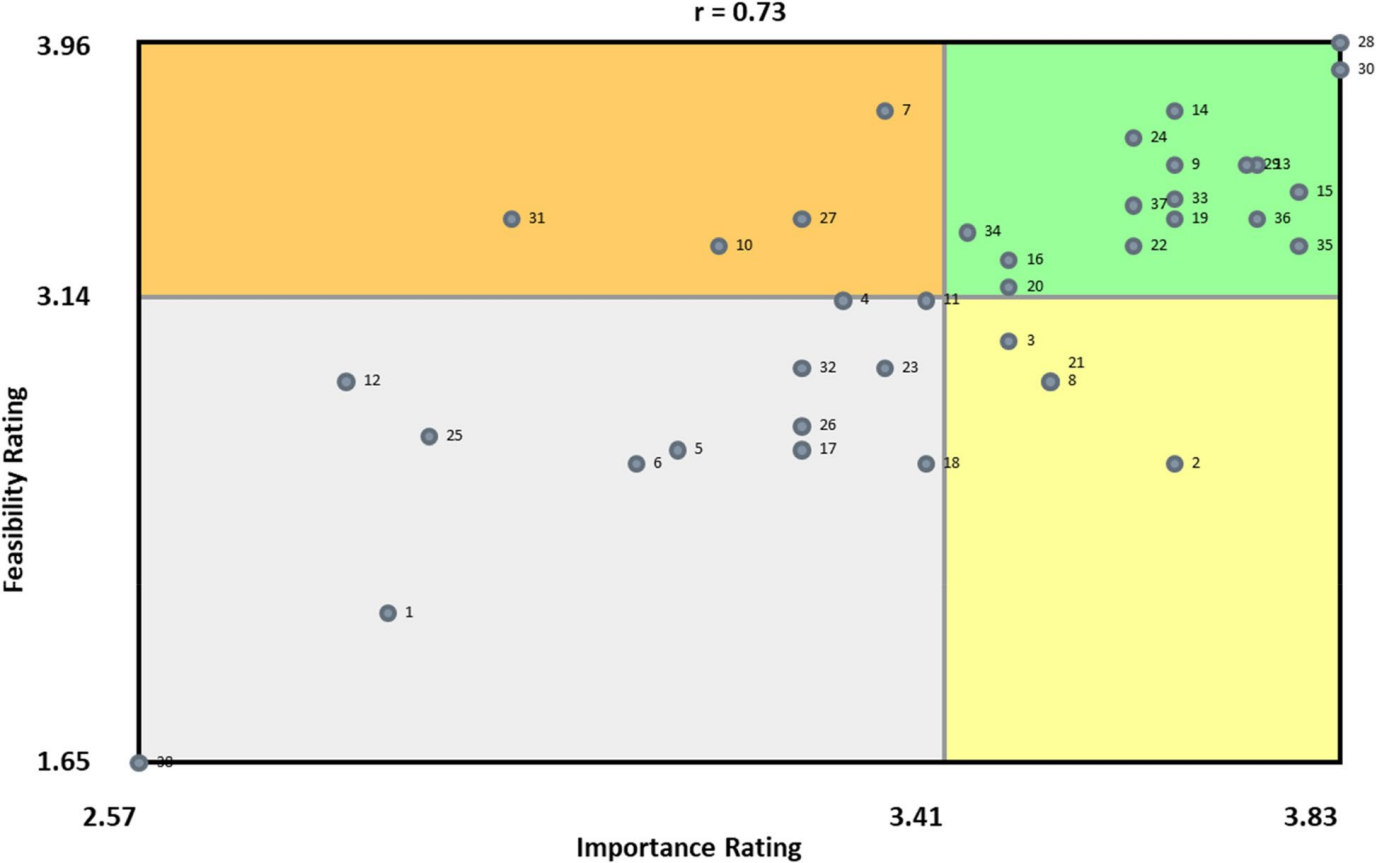
Individual EBS by feasibility and importance ratings (Go-Zone Map)

Two EBS from the “provider recommendation/communication” cluster emerged as the most important and feasible in the go-zone map: “Provide parents/adolescents an opportunity to ask about HPV vaccine during visits” (statement #28) and “Focus on HPV vaccines as a cancer prevention vaccine and not on sexual transmission” (statement #30) (Fig. 4). Two other EBS from the “nurse/staff workflow and training” cluster emerged as the next most important strategies in the go-zone map: “Set up EHR alerts for providers/staff to vaccinate adolescents due/overdue for the HPV vaccine” (statement #15) and “Clinic staff can pre-chart to see if the scheduled adolescent is due/overdue for HPV vaccine before each visit” (statement #35) (Fig. 4). Lastly, one EBS from the “reducing missed opportunities” cluster emerged as the next most feasible strategy within the go-zone map: “Encourage providers/staff to recommend HPV vaccine by bundling with other vaccines” (statement #14) (Fig. 4).

#### Comparison across participant groups

**Fig. 5:**
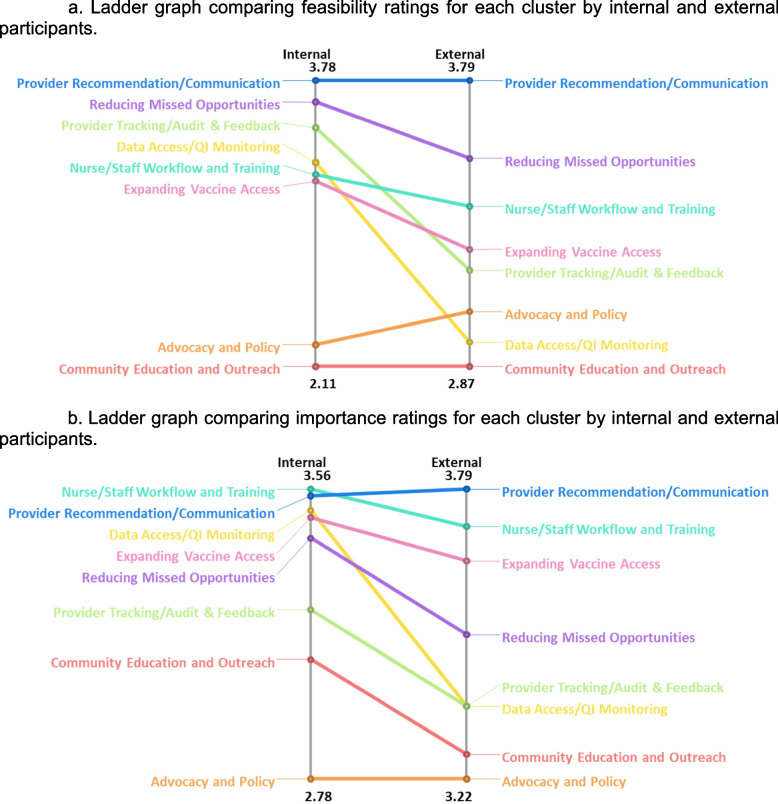
**a** Ladder graph comparing feasibility ratings for each cluster by internal and external participants. **b** Ladder graph comparing importance ratings for each cluster by internal and external participants

In our comparison of feasibility ratings between internal and external participant groups (Fig. 5a), both groups agreed on the top two most feasible clusters of EBS: “provider recommendation/communication” and “reducing missed opportunities.” When comparing ratings of importance across internal clinic and external community members **(**Fig. 5b**)**, internal clinic participants rated “nurse/staff workflow and training” as having higher importance than “provider recommendation/communication” strategies. Both internal and external groups continued to agree that both “nurse/staff workflow and training” and “provider recommendation/communication” were the top two most important clusters.

**Fig. 6:**
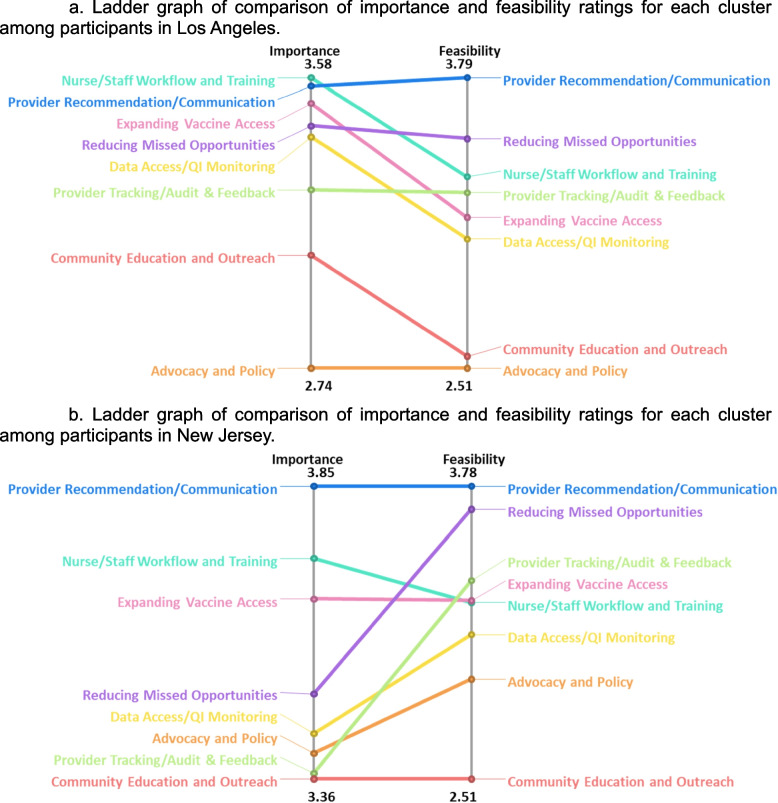
**a** Ladder graph of comparison of importance and feasibility ratings for each cluster among participants in Los Angeles. **b** Ladder graph of comparison of importance and feasibility ratings for each cluster among participants in New Jersey

We also compared ratings of importance and feasibility across regions (LA vs. NJ) (Fig. 6a and b) and observed consistent top-rated clusters for each rating (importance: “nurse/staff workflow and training” and “provider recommendation/communication;” feasibility: “provider recommendation/communication” and “reducing missed opportunities”). However, “nurse/staff workflow and training” had a higher mean importance rating in LA compared to the importance rating in NJ. “Community education and outreach” and “advocacy and policy” clusters were consistently rated among the least important and least feasible clusters across all participants and in analyses of subgroups, including comparisons of internal vs. external groups and the regional comparisons.

#### Interpretation findings

The interpretative session consisted of 6 participants (3 providers and 3 advocates) who took part in a 60-minute virtual discussion in September 2022. Participants reflected on how their role in HPV vaccination promotion influenced their sorting and rating decisions. Despite the diversity among participants in terms of their role in HPV vaccination and the communities that they serve (i.e., racial/ethnic marginalized communities, practice region), there was consistent agreement with the overall concept mapping results. For example, one advocate stated:

*“I think it definitely made sense putting them [in]to these different categories….I don’t think you missed anything if that’s what you’re asking….I’m not surprised to see it, let’s put it like that.”*

A provider also agreed that the final strategies in the go-zone map that were most important and most feasible aligned with their current practice:

*“I think what I see there just from my perspective as a pediatrician, I think that’s right… Our nurses are phenomenal and picking up those gaps and…our nurses will kind of pre-chart and see which patients are coming in and see what’s missing if this patient had gotten all their vaccines...So, the nurse and staff workflow is really important for us in terms of getting our kids vaccinated against HPV.”*

When the group was asked to interpret why the “community education and outreach” and “advocacy and policy” clusters were rated as some of the least important and feasible strategies, one provider stated:

*“I think people are thinking of the pandemic when much of the interaction that we would have in person in the community came to a screeching halt simply because we were not meeting. And not everybody [in the communities] can conduct Zoom meetings.”*

Importantly, one participant emphasized the need to be flexible with strategies to improve HPV vaccination and how the importance and feasibility of strategies will vary based on patient populations:

*“I think one has to not only adjust one’s activities in those clusters and how one speaks. It’s also the context in which we are speaking, who we are speaking with, and what do they have in that background that they bring to the table… one really has to be very nimble in moving from one cluster to the other. Both with time as well as in looking at the situation. As to whom one is speaking with and is it an organization, individual, parent, what.”*

#### Selection of strategies by FQHC leadership and physician champions:

FQHC system leaders and champions showed the most interest in eight strategies when presented with the concept mapping results. All eight strategies selected were in the green quadrant of the go-zone map (see Fig. 3), indicating most important and most feasible strategies based on ratings from all concept mapping participants. They selected three strategies from the “reducing missed opportunities” cluster: Normalize HPV vaccination as ‘opt-out’ event versus ‘optional vaccine’ during clinical encounter (statement #9), Encourage providers/staff to recommend HPV vaccine by bundling with other vaccines (statement #14), and Encourage providers/staff to recommend HPV vaccines assuming the parent/adolescents will receive it (announcement approach) (statement #24). They also selected two strategies from the “nurse/staff workflow and training” cluster: Set up EHR alerts for providers/staff to vaccinate adolescents due/overdue for the HPV vaccine (statement #15) an provide training for all clinic staff on HPV vaccine recommendation strategies to ensure consistent messaging (statement #37). Lastly, they selected the following three strategies from different clusters: “Monitor/report/provide feedback to individual providers on their HPV vaccine rates” (statement #16, provider tracking/audit and feedback cluster), “Focus on HPV vaccines as a cancer prevention vaccine and not on sexual transmission” (statement #30, provider recommendation/communication cluster), and “Provide opportunities for HPV vaccinations during all visits including sick/urgent care visits” (statement #33, expanding vaccine access cluster). We then delivered the eight selected strategies through physician trainings, clinic staff trainings, and audit and feedback meetings at each of the three clinics within the FQHC system during a 12-month intervention period following initial presentation of concept mapping results. Throughout the 12-month intervention period physician champions and system leaders were continuously engaged in monthly report back meetings and supported the recruitment of providers and clinic staff for intervention activities, indicating a participatory co-selection process using concept mapping may facilitate stronger engagement throughout the implementation process.

## Discussion

This is one of few studies that used concept mapping as a participatory approach to inform prioritization, selection, and fit of EBS for implementation in a large multi-site FQHC system [52–54]. Applying this approach to HPV vaccination within safety-net healthcare settings, we identified consistent ratings between internal and external groups in the high prioritization (importance and feasibility) of provider- and clinic-team focused strategies within the internal context for improving HPV vaccination for adolescents in safety-net clinics. Consistency across internal and external partners within the local context is important alignment in shared HPV vaccination change and in long-term sustainability of effective programs, supported by prior studies indicating user-centered designs are critical for implementation success [16]. Consensus among internal and external participants that communication tools for providers and staffing workflows were perceived as critical in addressing improved uptake has been found in other studies [55–57]. Further, the broader cancer prevention and control field has recognized translation of evidence-based tools into practice requires understanding the “dynamic, multilevel context” of both local/inner clinic and outer policy and communities contexts as well as working in partnership with clinical/community partners to co-create and co-design sustainable strategies [58, 59]. For HPV vaccination, specifically, while external advocates and researchers may prioritize data monitoring and provider specific audit and feedback, internal clinic members may rate these lower due to limited infrastructure, required resources, and competing priorities that limit the potential to fully implement such strategies. Without internal input, EBS that work in other healthcare settings may not be feasible in safety-net clinics or may require further adaptation for fit to the local context.

Our findings also support the ability of concept mapping to be used as a participatory engagement tool so that researchers and clinic systems consider, prioritize, and implement multilevel approaches [33, 60, 61]. We found that concept mapping can respond to the complexities of implementation at the inner and outer context by assisting in the prioritization and selection of EBS across internal and external groups for HPV vaccine improvement in safety-net clinics in general and for local clinic contexts, with respect to culture and values of specific communities and historical experiences with healthcare settings [62]. However, in comparing concept mapping findings across our two study regions (Greater LA and NJ), we did not find distinct differences in sorting, ranking, or other outcomes.

Our team identified the EBS for HPV vaccination, as individual statements, to be ranked and sorted based on strategies identified in our qualitative interviews and national guidelines. Our approach sought to provide a comprehensive set of EBS so that decision-makers might consider a broader set of possibilities than what they would generate with limited time. This modification to group concept mapping in the brainstorming phase has been utilized previously [39, 60, 63–65]. Furthermore, elicitation through qualitative interviews that were guided by the Practice Change Model [48], allowed for in-depth understandings of adaptations made in the context of COVID-19 pandemic constraints [66], which subsequently identified adaptations to be considered during the rating and sorting activities [39]. Interviews provided the opportunity for the research team to interact with respondents in order to ensure understanding of the prompt and request elaboration where constructs requested are not fully specified in original response [16, 39, 40, 65, 67]. For example, Kwok and colleagues speak to the benefit of being able to prompt respondents to associate a specific implementation strategy to the barriers that it might specifically seek to address. Moreover, we also used the same presentation on concept mapping findings for the concept mapping participant interpretation meeting and the multi-site FQHC participatory EBS selection process. Thus, the clinic system benefited directly from the ratings and prioritization of EBS by multilevel clinic and community partners within the broader safety-net clinic context [38].

Our findings also point to agreement across participants that community outreach, policy and advocacy, and data access were still viewed as important (e.g., average ratings of “somewhat important”) and feasible (e.g., average ratings between “not very doable right now” and “somewhat doable right now”) but prioritized below clinic-based strategies. While work by our team and others suggest the COVID-19 pandemic may have mobilized policy-level representatives and vaccine champions to identify new innovations in community outreach and local public health vaccination efforts that could be leveraged for HPV vaccination outreach, the implementation of such approaches has yet to be fully realized [15, 19]. This variation may be due to several factors. First, the participants may not have prioritized strategies outside of clinic settings in this study because our focus prompt emphasized improving HPV vaccination in “healthcare settings serving medically underserved communities [15].” Second, it may reflect the pandemic restrictions on community advocacy efforts, as pointed out by the participants in the interpretative session [47]. Lastly, these findings may reflect how responsibility is assigned for HPV vaccination, with a focus on internal clinic change rather than broader policy change.

While use of concept mapping facilitated collective prioritization of EBS for HPV vaccination across internal and external groups and directly informed local level implementation, some limitations should be noted. First, while we invited qualitative interview participants from the prior phase of this larger implementation study, not all participants from that phase participated in concept mapping. Only about 40 percent of participants returned for concept mapping. However, all participant groups from the qualitative interviews, aside from the healthcare payer group, were represented amongst the concept mapping participants. We also did not invite parent participants from the qualitative interviews and focus groups (n=7) that we conducted virtually in LA and NJ to participate in concept mapping because we focused on the adoption of clinic-level strategies within this phase of the larger study. Additionally, participants in this study represent two regions in the United States so our findings may not be generalizable to other areas [36]. While pre-specified strategies for HPV vaccination from qualitative interviews and guidelines were used in lieu of a brainstorming phase, which made for a more efficient process, not all EBS may have aligned with the concept mapping participants’ views. Lastly, our study was conducted between April and September 2022, when COVID-19 pandemic mitigation measures, vaccination efforts, and control efforts had not yet waned, therefore, our findings may be less generalizable to non-pandemic periods [68]. Our participants were also particularly aware of missed HPV vaccination opportunities and adolescent well-visits during the prior two years of the pandemic and may have rated specific strategies within the context of these issues [69, 70]. Nonetheless, findings from our study provide direction on acceptable and feasible EBS for HPV vaccination for moving forward in implementation within safety-net clinic settings in both regions as HPV vaccination activities resume [71].

## Conclusions

We identified consistency between internal and external groups in high prioritization of provider- and clinic-team focused HPV vaccination strategies, including increasing vaccine access, further supporting multilevel approaches to address equity. Our findings suggest concept mapping can respond to the complexities of implementation at the inner and outer context by assisting in the prioritization and selection of EBS across participant groups for HPV vaccine improvement. Concept mapping can elucidate requirements for EBS implementation, including those specific to settings that serve adolescents living in communities experiencing cancer inequities, and monitoring dissemination progress among key clinic and community partners. Further research is warranted in examining whether the entire concept mapping process or the concept mapping findings from a local community or region alone can be used to facilitate identification and adaptation of EBS for HPV vaccination in subsequent organizations and how concept mapping or other tools can facilitate sustainable participatory approaches to implementation and adoption of EBS for cancer prevention and control.

## Data Availability

The study materials used and data analyzed for this study are available from the corresponding author on reasonable request.

## Abbreviations

FQHC: Federally Qualified Health Center
HPV: Human Papillomavirus
EBS: Evidence-based Strategies
MA: Medical Assistant
RN: Registered Nurse
LVN: Licensed Vocational Nurse
CA: California
NJ: New Jersey
LA: Los Angeles
PCM: Practice Change Model
